# Applications of unmanned aerial vehicles in intertidal reef monitoring

**DOI:** 10.1038/s41598-017-10818-9

**Published:** 2017-08-31

**Authors:** Sarah L. Murfitt, Blake M. Allan, Alecia Bellgrove, Alex Rattray, Mary A. Young, Daniel Ierodiaconou

**Affiliations:** 1 Deakin University, School of Life and Environmental Sciences, Centre for Integrative Ecology, P.O. Box 423, Warrnambool, 3280 Victoria, Australia; 2 Victorian UAS Training, 57 Koroit-Woolsthrope Road, Koroit, 3282 Victoria, Australia

## Abstract

Monitoring of intertidal reefs is traditionally undertaken by on-ground survey methods which have assisted in understanding these complex habitats; however, often only a small spatial footprint of the reef is observed. Recent developments in unmanned aerial vehicles (UAVs) provide new opportunities for monitoring broad scale coastal ecosystems through the ability to capture centimetre resolution imagery and topographic data not possible with conventional approaches. This study compares UAV remote sensing of intertidal reefs to traditional on-ground monitoring surveys, and investigates the role of UAV derived geomorphological variables in explaining observed intertidal algal and invertebrate assemblages. A multirotor UAV was used to capture <1 cm resolution data from intertidal reefs, with on-ground quadrat surveys of intertidal biotic data for comparison. UAV surveys provided reliable estimates of dominant canopy-forming algae, however, understorey species were obscured and often underestimated. UAV derived geomorphic variables showed elevation and distance to seaward reef edge explained 19.7% and 15.9% of the variation in algal and invertebrate assemblage structure respectively. The findings of this study demonstrate benefits of low-cost UAVs for intertidal monitoring through rapid data collection, full coverage census, identification of dominant canopy habitat and generation of geomorphic derivatives for explaining biological variation.

## Introduction

Coastal regions are places of intrinsic value for the resources they provide, as well as being places of recreational and environmental importance^1^. With increasing pressures on coastal zones from climate change and coastal development, there is a need to effectively monitor these environments at scales capable of informing management decisions. Monitoring coastal habitats is multi-faceted, with a variety of data collection methods used in these environments, from traditional visual observational studies on intertidal and subtidal rocky reefs, and sandy beaches^2, 3^, to use of marine remote sensing technologies, such as underwater video monitoring, bathymetry from ship based sonar, and aerial based LiDAR approaches to characterise topographic structure and species distributions on subtidal reefs^4–6^. Fundamental to conserving marine biodiversity into the future is the understanding of natural spatial and temporal variability in intertidal reef biota and whether improved resilience to natural and anthropogenic disturbances may be achieved via management strategies, such as implementation of marine protected areas.

Intertidal reefs are dynamic and physiologically stressful ecosystems that can vary greatly in community structure over small spatial scales due to the mosaic of habitats created by biotic and abiotic influences^7^. Logistical constraints of monitoring intertidal reef biota during periods of emersion at low-tide and variable wave-exposure mean that replicated sampling of these areas can be challenging and time-consuming. Intertidal reef monitoring traditionally involves targeting small areas of the reef using quadrats, or along transects, often stratified by reef zonation, collecting data on species richness, abundance and community composition^8^. Although this provides an insight into community structure, it is labour intensive, captures only a small area of the reef under study, and generally does not collect information on the fine-scale variations in reef structure that may influence the distribution of assemblages observed. Gathering geomorphological data on the entire reef could assist with determining how differences in reef topography drive differences in species distributions across broader spatial scales and provide new insights into the spatial configuration of patterns observed.

Remote sensing of the earth and oceans has traditionally been performed by satellites and manned aircraft. Although these traditional methods of remote sensing can capture data over broad spatial scales there are major limitations when applied to monitoring intertidal reefs. For instance, the inability to provide high-resolution imagery required to map fine-scale heterogeneity (i.e. cm scale); inherently high costs associated with acquisition; limitations in terms of temporal collection due to orbits; and weather and associated constraints due to cloud cover^9^.

Unmanned aerial vehicles (UAVs), or drones, are beginning to bridge the disparity in scale between traditional remote sensing methods and on-ground monitoring techniques in various ecosystems^9^. The ability to collect remote sensing data on demand, using low cost platforms and sensors, provides new opportunities in the field of ecology to better understand patterns as well as processes driving them. Flying at lower altitudes (<100 m) than traditional remote sensing methods, allows for data capture below cloud cover, finer spatial resolution (sub cm) in outputs, and reduced costs^10^. These advantages allow for rapid collection of high-resolution data at precisely designed temporal scales^11^, which have seen UAVs emerge as an important tool in environmental conservation and monitoring^12, 13^.

The potential of UAVs for environmental assessment and monitoring is increasingly being demonstrated, and the impacts of environmental disturbances can be assessed rapidly because of the ease of deployment and portability of UAVs^14, 15^. Recent studies show the advantage of using UAVs in forest and crop monitoring^16, 17^, monitoring coastal erosion^15, 18, 19^, and quantification of submerged vegetation in shallow water^20^. UAV systems can also use multispectral sensors to assist with identification of species and vegetation health via spectral classifications^21^ and can be deployed to perform population censuses of species in remote or difficult to access areas^22–24^.

Whilst UAVs have been used to assess broad-scale coastal morphology along beaches and sand dunes^15, 19, 25^ applications to intertidal reef systems to investigate community structure have been limited, though a few studies have considered innovative ways to capture intertidal reefs at a much higher resolution using low altitude remote sensing platforms. For example, a blimp fitted with a multispectral sensor was deployed to map an intertidal reef from an altitude of 80 m to estimate algal biomass using normalised difference vegetation indices (NDVI) and topographic heterogeneity of the reef for scale-dependent analyses of algal-topography relationships^26^. Another study used a kite to collect imagery of a reef and construct high resolution (5 cm) 3D models to characterise the geomorphology of the reef and collect multispectral data over hundreds of metres^27^.

The ability to use low-cost multirotor platforms with autopilot systems provides distinct advantages for low altitude imagery platforms for intertidal reefs, including improved efficiency using pre-determined flight paths for data collection, critical to maximise survey time available at low tides. Off-the-shelf UAV platforms, easily accessible to the hobbyist, also provide the potential to enhance data output through citizen science programs, with low-cost platforms capable of autonomous programmed flight now common in the marketplace^12, 28^. This readily available technology will allow citizen scientists access to pre-programmed flight paths, smart ground control targets to allow for cm precision, and web-based workflows to automate data upload to central depositories for cloud processing and data dissemination. Over networks, this could provide scientists with high frequency image capture to monitor change not possible using scientific teams alone. Whilst yet to be fully exploited for citizen science, there is great potential for such UAV approaches to be effective in providing high frequency temporal data collection over targeted areas if adequate training and quality control can be provided, and flight regulatory requirements for small UAVs can be met^12^. There is also a need to quantify the detectability of species when using UAV surveys compared to traditional approaches to determine the value of low-cost UAVs to complement, or potentially replace, more field-intensive ground based approaches. Through accurate geo-referencing of imagery mosaics, UAVs provide the potential to identify subtle shifts in species distribution with repeat surveys. In addition, digital surface models from UAV surveys make it possible to collect data on the variation in geomorphic features, such as subtle changes in elevation and complexity that have been found to influence biotic assemblages^29^, and susceptibility to sea level rise with a changing climate^30^.

This study assesses the potential for UAVs to accurately monitor intertidal reefs. This was achieved by comparing dominant algal communities measured using on-ground quadrat surveys to those extracted from UAV captured photogrammetry. Using fine scale geomorphic features derived from UAV data capture, environmental drivers of biological variation on the reef are also determined. UAV geomorphic features representing variations in reef structure were compared to species counts from on-ground quadrat surveys to determine their influence on intertidal macroalgae and invertebrate assemblages observed. Automated unsupervised classification of canopy-forming macroalgae was also tested to explore the potential for full intertidal reef census.

## Methods

### Study Sites

**Figure 1:**
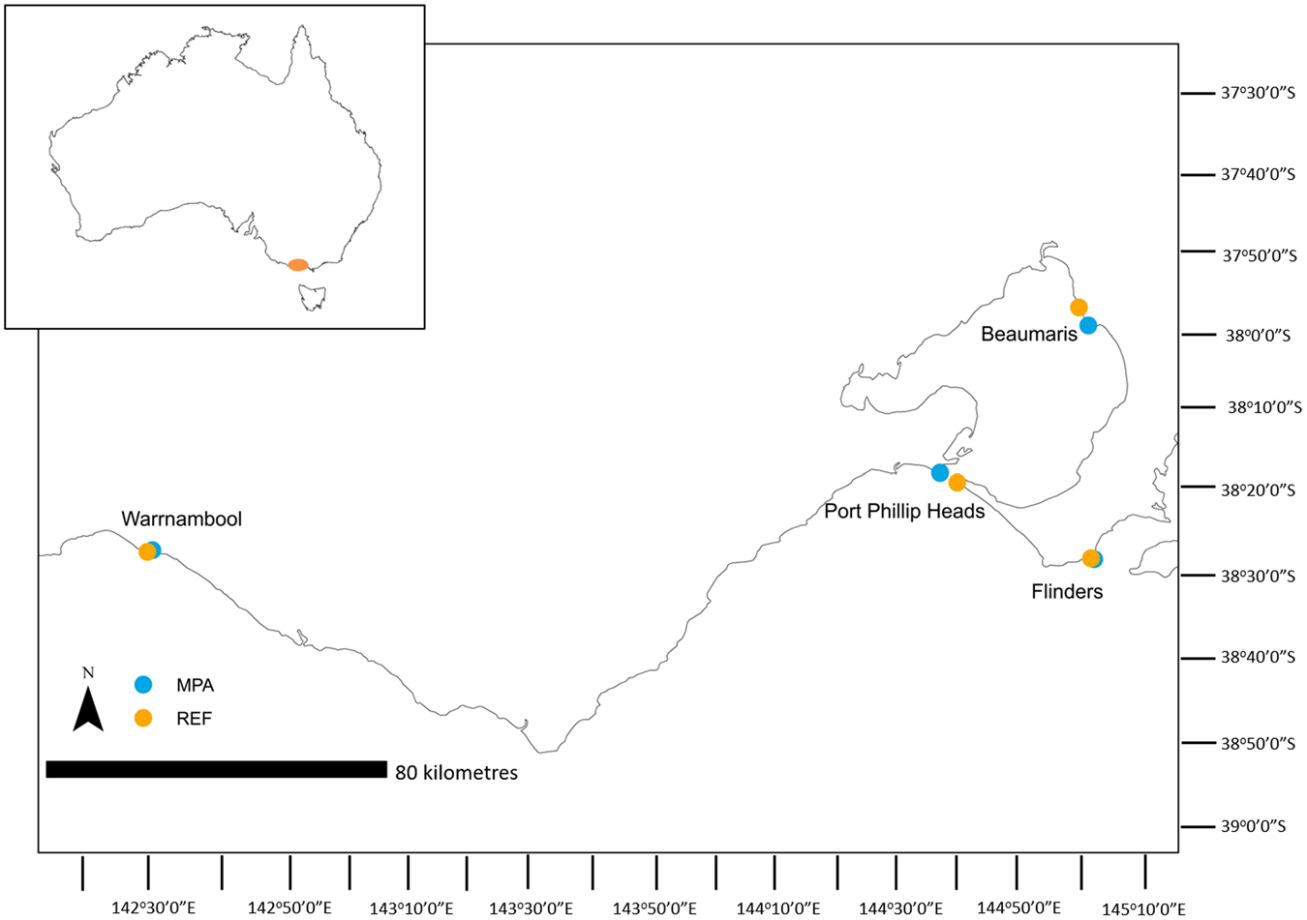
Study sites of the four regions along the coast of Victoria, Australia with sites inside marine protected areas (MPA) and reference sites outside MPAs (REF) identified. Inset shows study area on the south-east coast of Australia. Maps created in ArcMap (v.10.4.1; esri.com).

Study sites were identified from the Parks Victoria Intertidal Reef Monitoring Program to represent intertidal reefs from open coast to embayments along a longitudinal gradient. Four regions (Fig. 1) were chosen along the Victorian coast, each containing a site in a marine protected area (MPA) and an unprotected reference site (REF). The sites were Pickering Point (MPA: −38°24′S, 142°28′E) and Shelly Beach (REF: −38°23′S, 142°27′E); Point Lonsdale (MPA: −38°17′S, 144°36′E) and Cheviot Beach (REF: −38°18′S, 144°39′E); Ricketts Point (MPA: −37°59′S, 145°01′E) and Halfmoon Bay (REF: −37°58′S, 145°05′E); and Mushroom Reef (MPA: −38°29′S, 145°01′E) and West Flinders (REF: −38°28′S, 145°06′E) (Fig. 1). The low-tide range for surveys was between 0.12–0.26 m, and were in a semi-diurnal tidal regime.

### UAV surveys

The UAV platform used was a Swellpro Splashdrone quadcoptor (http://www.swellpro.com), 32 cm wide with a height of 25 cm, and a weight of 2.5 kg when fully equipped. The UAV was fitted with an open source Ardupilot (http://ardupilot.org) flight control system to allow for autonomous flight along pre-prepared flight paths. The sensor used was a waterproof Canon D30 (https://www.canon.com) with a resolution of 4592 × 3056 pixels and an automated trigger system, which captured an image every 2 seconds. The entire airframe and payload is waterproof for use in coastal and marine environments, to allow for a safe emergency landing in the ocean if required.

#### UAV data acquisition

Mission Planner software (v. 1.3.35) was used to prepare flight paths for each site, and monitor progress during UAV data capture. Flight missions were flown at an altitude of 10 m above Australian Height Datum (AHD) and a speed of 2 ms^−1^. UAV flight paths were designed in a cross-hatch pattern to allow for greater overlap in imagery, with a 60% overlap and 40% sidelap to the cross-track design used to ensure high data redundancy. For larger sites, multiple flights were required due to limited battery endurance (15 minutes). Surveys were carried out on days with low wind speed (<15 knots) and low tides to ensure the greatest spatial capture of the exposed intertidal reef. UAV flights were undertaken immediately after the tide had receded to maximise coverage that could be achieved before the incoming tide, and low-tide times were selected to avoid glare from the high sun close to midday.

Black and white, 30 × 30 cm checkerboard targets were deployed at each site as ground control points (GCPs) for accurate geo-referencing of imagery. The centre of these targets was recorded using a Topcon (https://www.topconpositioning.com) Hiper-S real time kinematic global positioning system (RTK GPS) with <2 cm precision for latitude, longitude, and elevation achieved by streaming real-time corrections via the 4 G cellular network from the GPSNET base station network (http:/gnss.vicpos.com.au).

#### UAV processing

**Figure 2:**
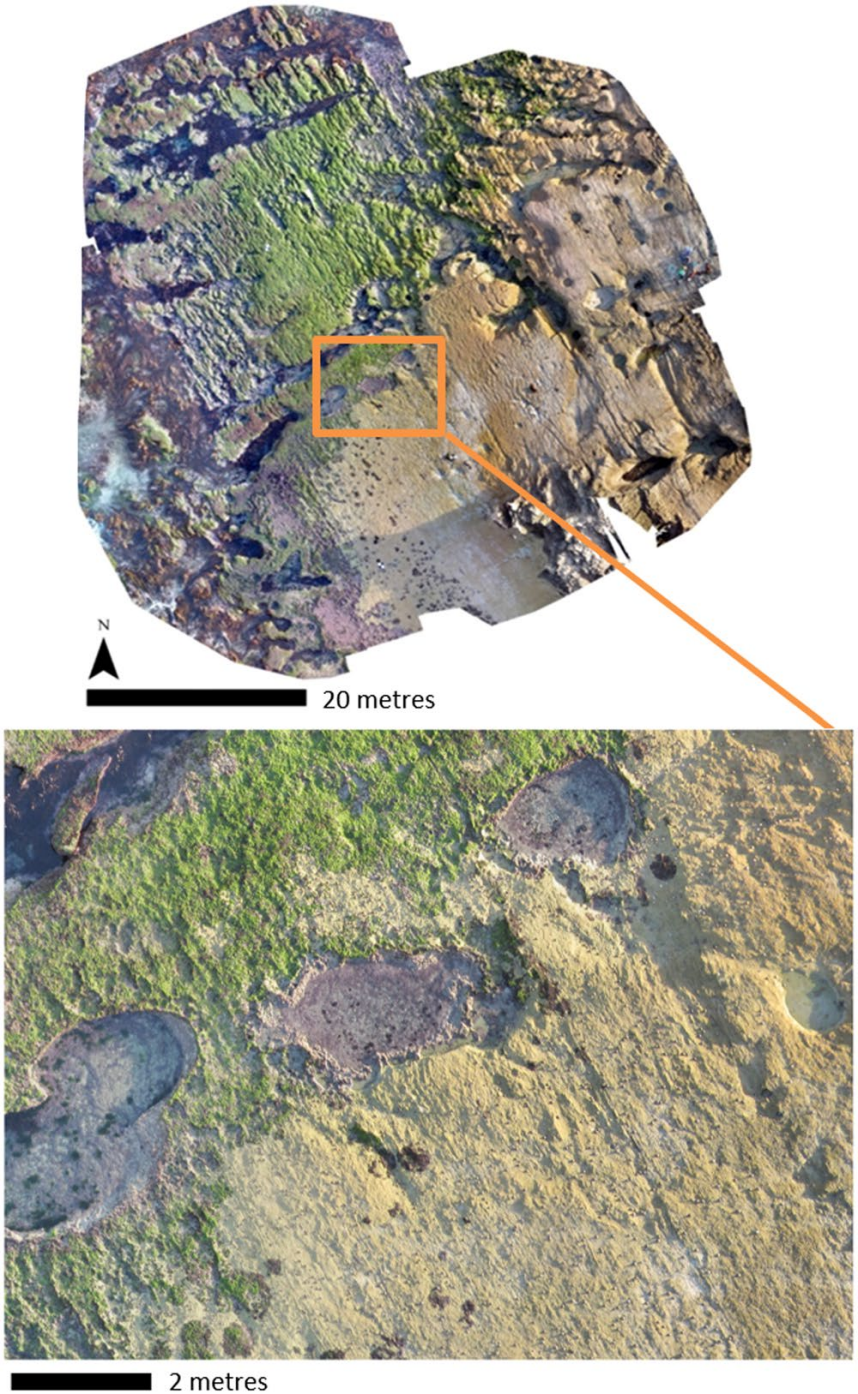
Orthomosaic of Shelly Beach site in Warrnambool, inset showing detailed section of the reef, derived from 10 m altitude unmanned aerial vehicle (UAV) flight.

Images collected from UAV flights were geotagged with Mission Planner (v. 1.3.35) prior to photogrammetric processing using Pix4Dmapper software (v. 2.1.53). GCPs were added to each project, and manually tied to several images, to produce a geo-referenced orthomosaic. This allowed for vertices of the on-ground quadrats recorded with RTK GPS to be located with high precision for extraction of UAV virtual quadrats. Full processing was initiated in Pix4Dmapper software (v. 2.1.53) which searched for matching points in uploaded images and calibrated the position and orientation of image capture; calculated 3D coordinates of images to create a point-cloud; and generated a digital surface model (DSM) and geo-referenced orthomosaic for each of the eight sites (Fig. 2).

**Figure 3:**
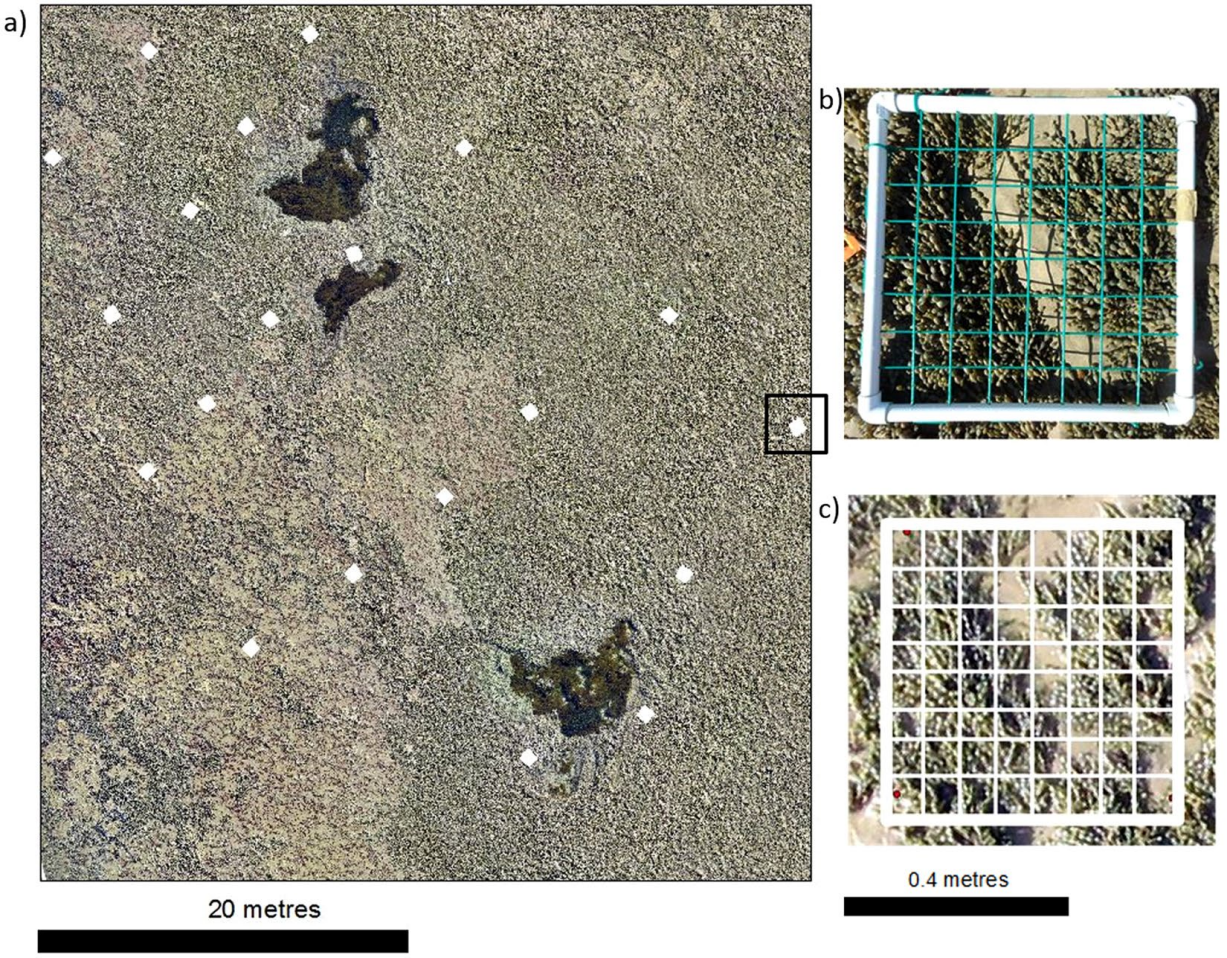
(**a**) Section of Cheviot Beach orthomosaic with UAV virtual quadrat positions collected from corners of on-ground quadrats, (**b**) photo of on-ground quadrat, and (**c**) extracted UAV virtual quadrat with point count grid overlay. Dominant canopy forming alga Hormosira banksii is seen throughout **a**–**c**.

Orthomosaics for each site were imported into ArcMap (v. 10.4.1) along with RTK GPS data points from the coordinates of on-ground quadrat corners to allow for data co-location. UAV virtual quadrats were exported into Coral Point Count software (v. 4.1) and a 50-point grid created to remotely sense percentage cover of dominant algae (Fig. 3). After preliminary UAV surveys at two of the sites, it was decided to classify the algal taxa into four dominant groups (*Hormosira banksii*, turfing rhodophytes, chlorophytes, and encrusting corallines), due to similar morphology of some species, limitations of the sensor applied, and in some cases, insufficient resolution to confidently differentiate species.

Secondary products were derived from the DSM to characterise the structural complexity of the intertidal reef likely to influence biotic assemblages observed. DSM resolution for all sites was resampled to 5 cm in the horizontal plane from an average output resolution of 0.29 cm for comparable analysis and to reduce the influence of micro roughness by fine scale biological components, such as macroalgae. Environmental variables were derived from the UAV virtual quadrats using Benthic Terrain Modeller 3.0 in ArcMap (v. 10.4.1). The mean elevation, vector ruggedness measure (VRM), aspect (northness and eastness), and distance to seaward reef edge, were calculated for each quadrat at all sites using a 3 × 3 kernel.

### On-ground quadrat surveys

On-ground quadrat data were collected in accordance with the Parks Victoria Intertidal Reef Monitoring Program, and where possible completed during the same tide as the UAV surveys. At each site 25 existing fixed-position quadrats were surveyed along five transects running from the high- to low-shoreline using a 0.25 m^2^, 50-point quadrat, with percentage covers of algae and sessile invertebrates, abundances of mobile invertebrates, and photo-quadrats recorded. A total of 198 quadrats were surveyed over the eight sites, with 25 quadrats surveyed at each site, except Point Lonsdale and Mushroom Reef (*n* = 24 quadrats per site) due to restricted low-water time resulting in missing an individual quadrat at each location. Algae and invertebrates were identified to the lowest possible taxonomic level. Coordinates of quadrat corners were recorded with RTK GPS for co-location within the UAV orthomosaic.

### Automated macroalgal classification

Automated classification was tested using ISO Cluster Unsupervised Classification in ArcMap (v. 10.4.1) on the Point Lonsdale site using the dominant fucoid *H. banksii*, to test the potential for full reef census. A section of the site (area = 2935.74 m^2^) was selected for the analysis and resampled to 1 cm resolution to assess the potential resolution of higher altitude flights. Fifty 0.25 m^2^ virtual quadrats were randomly selected to quantify percentage cover using manual and ISO image classification approaches. Percentage cover of the manual and ISO automated quadrats were then extrapolated to estimate percentage cover of the section, and compared with full coverage ISO image classification for the section to determine the potential of upscaling percentage cover estimates from traditional survey approaches. Percentage cover of manually and automatically classified *H. banksii* cover in the 50 virtual quadrats was compared by linear regression and paired t-tests in R statistical software^31^.

### Comparison of assemblage data collected by UAV and on-ground quadrat surveys

Algal assemblage groups were compared among regions, parks, and methods using a mixed-model permutational multivariate analysis of variance (PERMANOVA) in PRIMER (v. 7.0.11) with the PERMANOVA+ add on. Data were square-root transformed to account for variation in species abundances and prevent undue influence from a small number of very common taxa^32^, and then tested with unlimited permutations of data, using Bray-Curtis similarity matrices^33^. The statistical design had four crossed factors: Region (four levels, random); Park (two levels: MPA and reference, fixed); Method (two levels: on-ground quadrats and UAV, fixed); and Quadrat (twenty-five levels, fixed). Tests for homogeneity of dispersions within factors were performed using PERMDISP^34^ with distance to centroids. Pairwise analyses were conducted between on-ground and UAV virtual quadrat methods for significant interaction terms, and similarity percentages (SIMPER) calculated to determine species contributing the greatest to the dissimilarities between survey methods. Non-metric multi-dimensional scaling (nMDS)^35^ was used to visualise the differences in assemblages detected using the two survey methods. Ordinations were accepted if stress values were <0.20^32^. Bray-Curtis resemblance data was verified through Shepherd diagrams^34^. Time taken to complete all on-ground quadrats was compared to time taken to complete UAV survey, extraction of virtual quadrats and virtual quadrat analysis combined through paired t-tests in R statistical software^31^. Time was calculated from the commencement of the first, to the completion of the final quadrat for on-ground counts; and UAV surveys were calculated by flight time combined with time taken for extraction of UAV virtual quadrats, and algal percentage cover analysis in Coral Point Count software (v. 4.1). Processing times for UAV data capture were not included, as after initial upload of data this process is semi-autonomous.

### Geomorphological and environmental influence on assemblage structure

Environmental variables were checked for correlation in PRIMER through Draftsman plots, and measure of multicollinearity checked by variance inflation factor (VIF) with all variables used showing VIF values < 5. Environmental variables were then normalised to account for variation in units, and their influence on intertidal community assemblages tested with BIOENV analyses. BIOENV tests were run to address the influence of MPA and reference sites on assemblage structure. To determine the percentage contribution environmental variables had on biotic assemblages, distance-based linear model multivariate analysis (DistLM) was performed. Distance-based redundancy analyses (dbRDA) with environmental variable overlays were used to visually analyse the influence of environmental variables on assemblage structure across the sites^36^.

## Results

Digital outputs created from the UAV imagery had an average resolution of 0.29 ± 0.01 cm; and average geo-location accuracy of 0.61 ± 0.18 cm, 0.65 ± 0.24 cm, and 1.19 ± 0.42 cm for latitude, longitude and altitude, respectively, for the eight sites (Table 1).

**Table 1:** Information from the Pix4Dmapper processing of all eight UAV surveys, including the number of ground control points (GCPs) used at each site for geo-referencing and RMS error in X, Y, and Z axes of the GCP locations.

	Number of calibrated images	Ground resolution (cm)	Area (hectare)	Number of GCPs	RMSE X (cm)	RMSE Y (cm)	RMSE Z (cm)
Pickering Point	256	0.34	0.726	4	0.007	0.228	0.390
Shelly Beach	144	0.33	0.344	4	0.393	0.376	1.082
Point Lonsdale	950	0.26	1.438	3	0.202	0.483	0.967
Cheviot Beach	564	0.28	0.883	8	1.445	2.283	3.819
Ricketts Point	325	0.25	0.446	4	0.718	0.329	0.638
Halfmoon Bay	200	0.27	0.149	5	0.669	0.492	1.812
Mushroom Reef	268	0.29	0.789	5	0.290	0.481	0.132
West Flinders	270	0.27	0.583	5	1.187	0.539	0.684

UAV surveys took significantly less time to complete than on-ground quadrat surveys (t = 8.757, P = <0.001). Although the UAV remote sensing only quantified a single stratum of algal cover, the results show total UAV time was approximately half that of the on-ground survey time (Supplementary Table S1), although greater detail was observed in the data collected from the on-ground quadrats. UAV flights took an average of 13.7 ± 4.8 minutes to complete plus 49.9 ± 4.4 minutes post-processing per site (total 63.6 ± 5.0 minutes per site), whilst the average time to complete all on-ground quadrats at a site was 126.3 ± 8.1 minutes.

Reliable identification of intertidal biota from UAV virtual quadrats was limited by image resolution and canopy-forming species obscuring the understorey. Consequently, invertebrate taxa were excluded and alga taxa were classified into four major groups (*Hormosira banksii*, turfing rhodophytes, chlorophytes, and encrusting corallines) for comparisons between on-ground quadrat and UAV virtual quadrats. The differences in the algal assemblages detected by the two methods were not consistent between sites inside marine protected areas (MPAs) and reference sites due in part to differences in dispersion between groups (PERMANOVA Park × Method interaction: Pseudo-F_(3,395)_ = 55.49, P_(perm)_ = <0.001; PERMDISP F_(3,392)_ = 8.583, P_(perm)_ = <0.001; Supplementary Table S2, Supplementary Fig. S1). Similarly, the differences in the composition and percentage covers of algal groups between sites inside marine protected areas (MPAs) and reference sites were not consistent amongst regions (PERMANOVA Region × Park interaction: Pseudo-F_(3,395)_ = 101.07, P_(perm)_ = <0.001; PERMDISP F_(7,388)_ = 40.58, P_(perm)_ = <0.001; Table 2, Supplementary Table S2, Supplementary Fig. S1)

**Table 2:** Mean percentage cover (±SE) of on-ground quadrats and UAV remotely sensed virtual quadrats, across four major algal groups for each site. All sites had 25 quadrats, except Point Lonsdale and Mushroom Reef (*n* = 24). Sites within marine protected areas highlighted in bold.

	On-ground Quadrats				UAV Virtual Quadrats			
	Hormosira banksii	Turfing rhodophytes	Encrusting corallines	Chlorophytes	Hormosira banksii	Turfing rhodophytes	Encrusting corallines	Chlorophytes
Pickering Point	**3.36 ± 1.55**	**15.56 ± 2.40**	—	**0.92 ± 0.30**	**3.76 ± 1.90**	**23.84 ± 2.79**	—	**1.56 ± 0.57**
Shelly Beach	—	17.36 ± 4.04	1.04 ± 0.74	20.44 ± 3.84	—	5.8 ± 2.01	—	14.12 ± 2.61
Point Lonsdale	**37.92 ± 2.39**	**0.88 ± 0.55**	—	**0.38 ± 0.33**	**37.17 ± 2.55**	**0.17 ± 0.16**	—	**0.08 ± 0.08**
Cheviot Beach	34.48 ± 3.08	12.40 ± 1.90	0.60 ± 0.28	5.52 ± 1.57	34.64 ± 3.29	2.48 ± 0.94	—	—
Ricketts Point	**8.32 ± 2.33**	**0.16 ± 0.12**	—	**2.04 ± 0.83**	**10.52 ± 2.92**	**0.28 ± 0.12**	—	**0.32 ± 0.15**
Halfmoon Bay	—	7.76 ± 1.84	—	0.64 ± 0.39	—	5.12 ± 1.39	—	—
Mushroom Reef	**3.42 ± 1.49**	**0.38 ± 0.16**	**0.29 ± 0.29**	—	**2.75 ± 1.11**	**0.21 ± 0.14**	**2.96 ± 1.40**	—
West Flinders	11.12 ± 1.94	19.92 ± 3.11	0.80 ± 0.29	0.08 ± 0.08	8.76 ± 1.98	3.96 ± 0.92	0.20 ± 0.10	—

Pairwise analysis showed no significant difference in algal assemblages between on-ground and UAV virtual quadrat methods at MPA sites (t = 0.489, P_(perm)_ = 0.710), although a significant difference between the two methods was observed for reference sites (t = 12.164, P_(perm)_ = 0.001). SIMPER analysis indicated that the dissimilarity in assemblages between methods at reference sites was explained by turfing rhodophytes (39.6%) and *H. banksii* (35.4%).

### Geomorphological and environmental influence on assemblage structure

Elevation and distance to seaward reef edge were the most influential environmental features, with elevation as the best explanation for algal taxa occurrence (ρ = 0.17), and a combination of elevation and distance to seaward reef edge explaining the most variation for invertebrate assemblages (ρ = 0.31).

**Figure 4:**
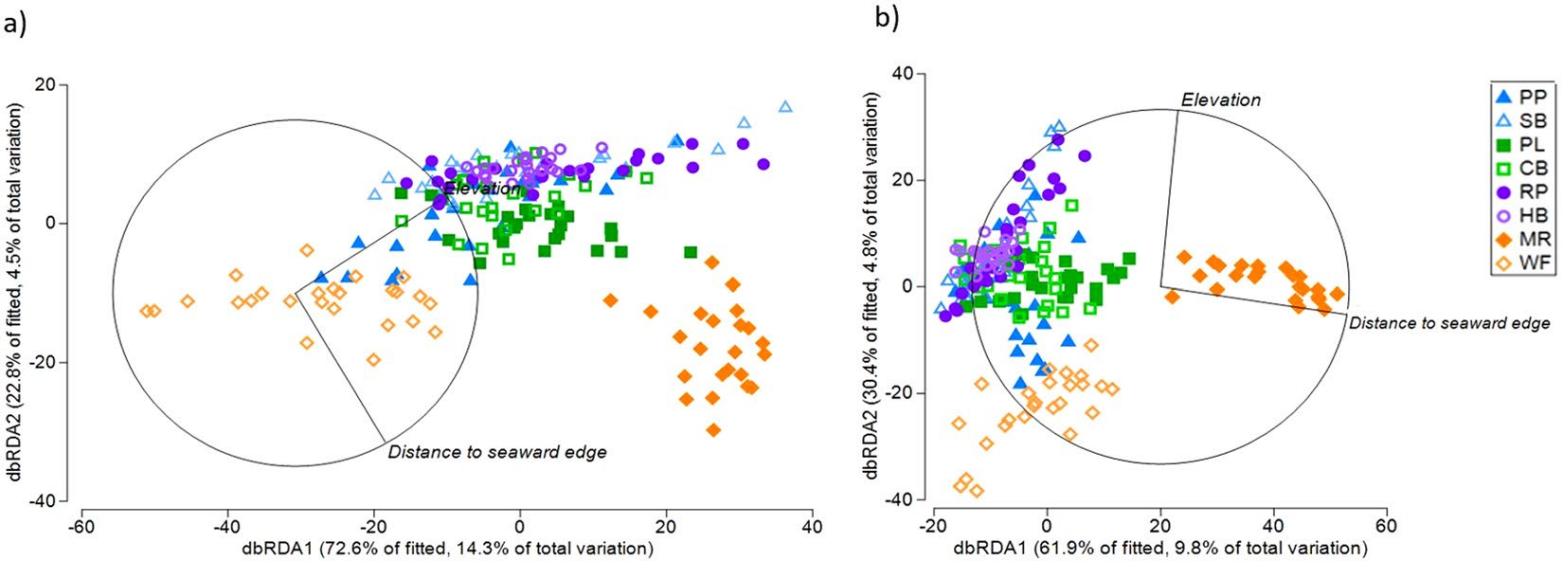
Distance-based redundancy analysis (dbRDA) ordination describing the relationship between UAV derived environmental variables and (**a**) algal percentage cover, and (**b**) invertebrate abundances from on-ground quadrat DistLM classified by site. Vectors depict the effect of environmental variables influencing presence of algae and invertebrates, with length of vector representing the strength of effect. Axes show percentage of variation: (**a**) elevation (dbRDA1) and distance to seaward reef edge (dbRDA2), and (**b**) distance to seaward reef edge (dbRDA1) and elevation (dbRDA2). Sites: (PP = Pickering Point, SB = Shelly Beach, PL = Point Lonsdale, CB = Cheviot Beach, RP = Ricketts Point, HB = Halfmoon Bay, MR = Mushroom Reef, WF = West Flinders); Park (MPA = closed, reference = open).

Distance-based linear models (DistLM) showed that 19.7% and 15.9% of the total variation in assemblage composition was attributable to environmental derivatives for algae and invertebrates, respectively. Elevation explained the greatest percentage of variation (14.9%) in the algal assemblages (*r* = 0.799, P = 0.001; Fig. 4), with seaward reef edge explaining 4.5% of the variation (*r* = −0.860, P = 0.001; Fig. 4). Invertebrate assemblage variation was principally explained by distance to seaward reef edge (9.8%; *r* = 0.982, P = 0.001; Fig. 4) and elevation (4.8%; *r* = 0.991, P = 0.001; Fig. 4). Vector ruggedness measure (VRM) was also significant for invertebrate assemblages (*r* = −0.928, P = 0.023), however only contributed 0.7% of the total variation^36^.

### Automated macroalgal classification

**Figure 5:**
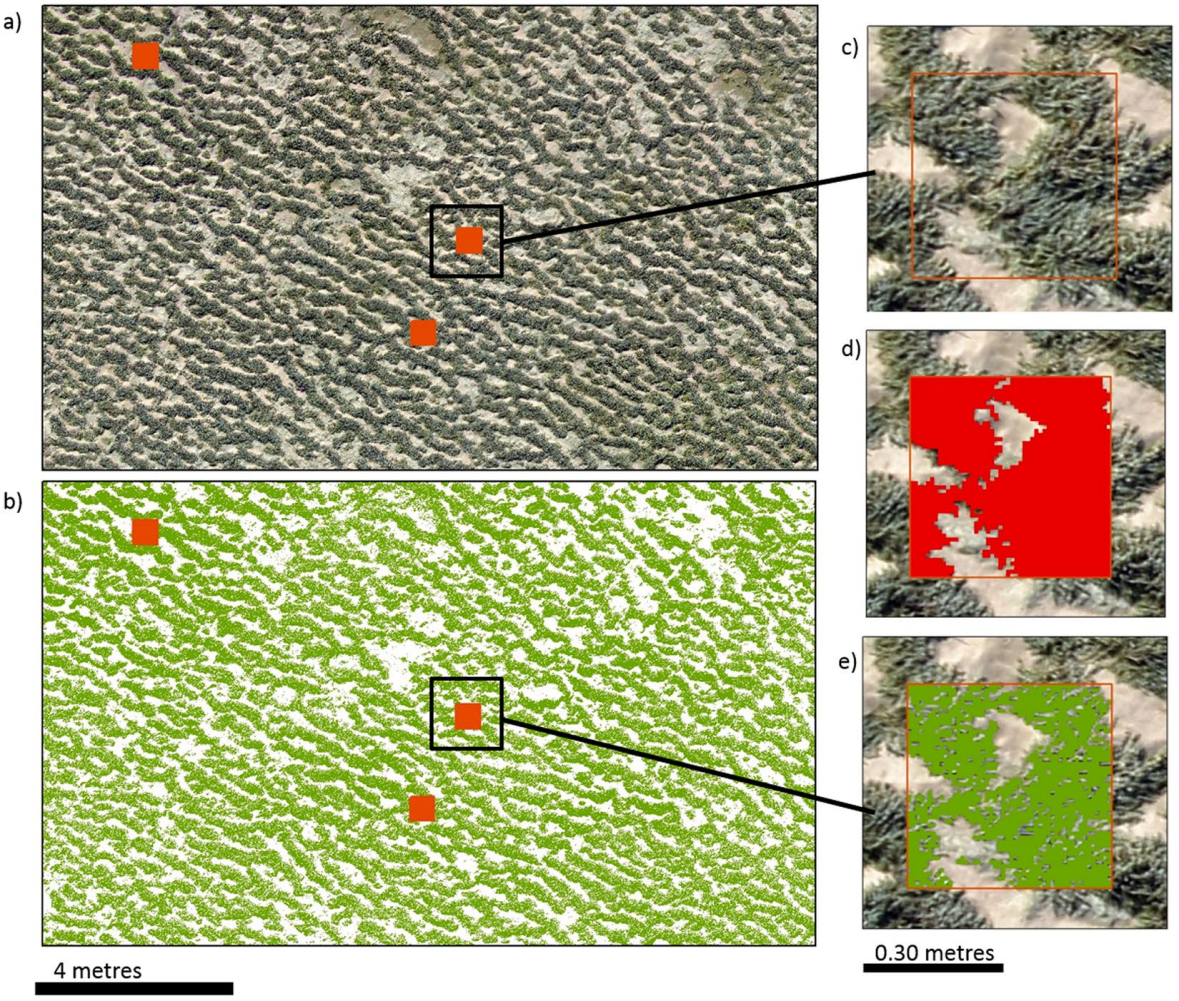
(**a**) section of Point Lonsdale site showing 0.25 m2 quadrats used for automated classification, (**b**) same section shown as ISO unsupervised classification with *Hormosira banksii* represented in green, (**c**) one of the 50 random quadrats across the site, (**d**) manual classification of *H. banksii* (red) for comparison with automated classification, (**e**) ISO classification of *H. banksii* (green).

Linear regression showed a significant positive relationship between the manual and ISO automated classification of *H. banksii* cover derived from the UAV imagery for Point Lonsdale (R^2^ = 0.64, P = <0.001; Supplementary Fig. 2). However, there was a significant difference in the percentage cover of *H. banksii* between the manual and ISO automated classifications (Paired t-test t = 3.866, P = <0.001), with the automated classification underestimating *H. banksii* cover by approximately 27%. Extrapolation of *H. banksii* cover from the 50 virtual quadrats to the clipped section of the Point Lonsdale site was 57.95% for manually classified and 46.95% for ISO cluster unsupervised classification (Fig. 5). The extrapolation of the 50 ISO virtual quadrats resulted in the same percentage cover as the ISO classification of the entire clipped section (46.95%). Cover of *H. banksii* from on-ground quadrat surveys in the section (*n* = 14) were also extrapolated to the clipped section (63.71%).

## Discussion

Use of UAVs in the present study reveals the potential for high-resolution remote sensing to be implemented into current intertidal monitoring efforts through simultaneous estimation of algal canopy cover and quantification of fine-scale geomorphological attributes of entire intertidal reefs. The present study expands on knowledge gained from on-ground surveys to encompass broader spatial scales, giving insight into the distribution and abundance of biota across intertidal reefs. Important findings include the quantification of dominant, canopy-forming macroalgae from the UAV imagery similar to on-ground measures; the ability to capture fine (cm) scale geomorphological variables of the whole reef; and the potential of upscaling to a full reef census with automated classification approaches. Although studies have applied aerial photogrammetry at centimetre resolution on intertidal^26, 27^, the present study builds on these through the application of autonomous flight; and the collection of precise, geo-referenced on-ground data to enable methodological comparisons.

Results showed no significant difference between UAV and on-ground methods for macroalgal canopy cover at MPA sites, driven by dominance of the fucoid *Hormosira banksii*. The ability to easily identify the *H. banksii* canopy in this study shows an advantage of utilising UAVs in the monitoring of intertidal reefs, as canopy-forming alga are often associated with other intertidal biota and shifts of these species could influence the species composition on the reef ^37^. Quadrat surveys provide very thorough observations of targeted sections of the reef but fail to capture the complete interconnected reef due to often limited emersion times and size of some intertidal reefs. The results reveal a difference in total time taken to complete the survey of the intertidal reef, with UAV survey and analysis taking approximately half the time of on-ground quadrat observations. Extrapolation of *H. banksii* from the on-ground quadrats showed larger estimates of percentage cover when extrapolated to the clipped section at Point Lonsdale than the ISO automated classification for the area; however, this might have been influenced by quadrat placement at the site. Quadrat placement along a stratified habitat design, may allow for a more balanced estimate when extrapolating to the entire reef. Estimations may also improve through machine learning techniques and classification, and the use of multispectral sensors to assist with discriminating between biota as well as opportunities for evaluating condition through vegetation indices. Conversely, more sophisticated sensors will incrementally increase costs associated with UAV surveys.

**Figure 6:**
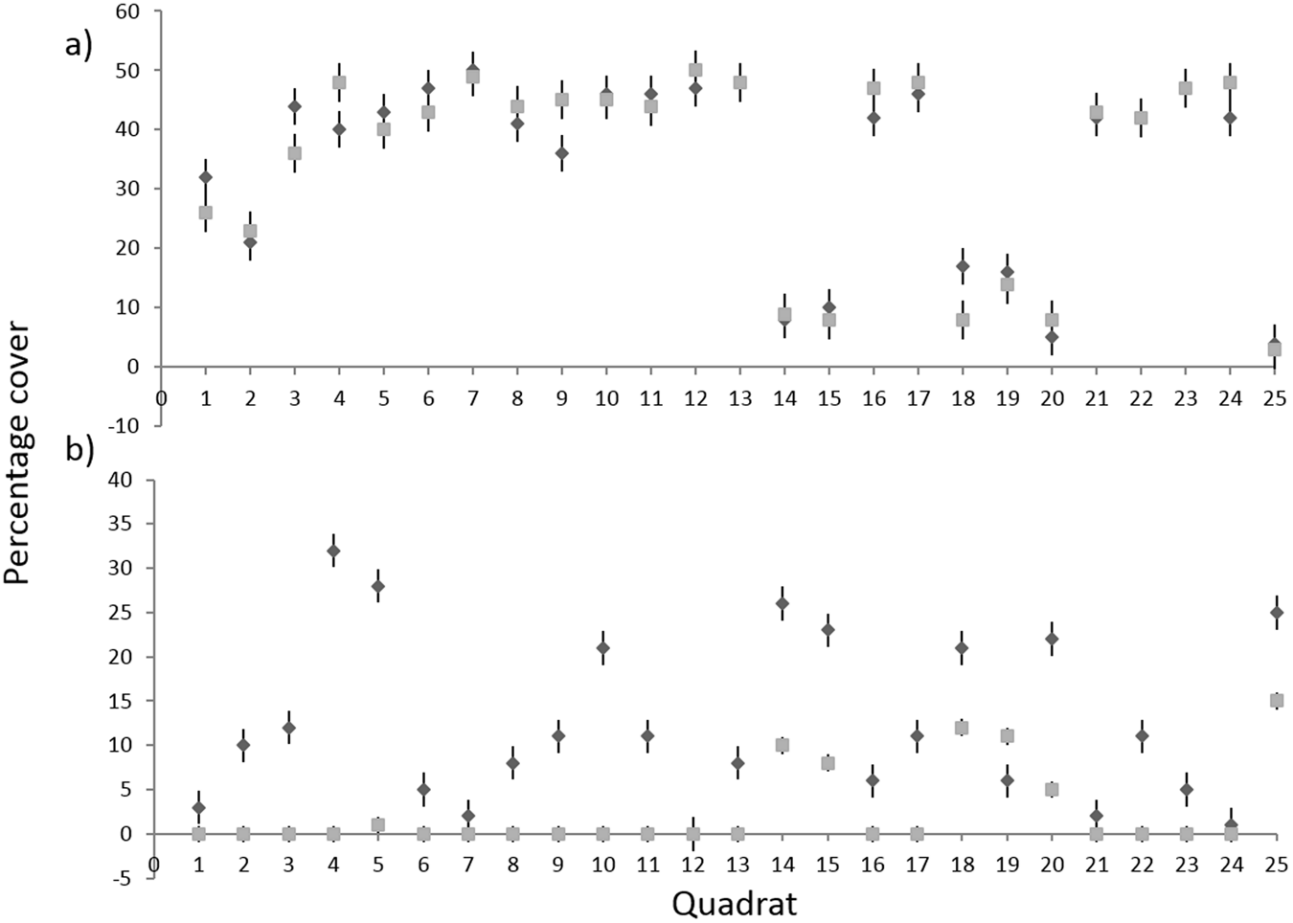
Comparison of broad group algal percentage cover recorded by on-ground quadrats (dark grey) and manual classification of UAV virtual quadrats (light grey) across 25 quadrats at Cheviot Beach site: (**a**) canopy-forming *Hormosira banksii*, (**b**) understorey turfing rhodophytes.

Dominant macroalgae and habitat heterogeneity were easily quantified in the UAV imagery, however, a limitation of the UAV quantification was the inability to observe biota under the canopy cover. Canopy-forming algae are common constituents of undisturbed intertidal reefs around the world^38, 39^, and it would be expected that the understorey biota would be obscured in the UAV surveys. The sites in the present study had a dominant single-species canopy cover, *H. banksii*, however, the ability to categorise multi-species canopies accurately may depend on the sensor resolution, the morphological distinctions between species visible in the UAV images, and the degree of vertical stratification of the canopy. Multispectral sensors may provide greater opportunity for spectral differentiation for multi-species canopies. Results showed a significant difference between percentage cover estimates from on-ground and UAV virtual quadrats for four algal groups, which may have been driven by dominant canopy cover obstructing understorey algal species. SIMPER analysis showed that the main driver of the difference between methods at reference sites was the turfing rhodophyte group, comprised primarily of *Capreolia implexa, Ceramium flaccidum*, and *Corallina officinalis*. These three species had low mean percentage covers across sites (*C. implexa* 3.9% ± 0.5; *C. flaccidum*. 1.4% ± 0.4; *C. officinalis* 3.9% ± 0.5) and may have been obscured in the understorey^37, 40, 41^ making them less visible in UAV virtual quadrats. As this algal group had greater percentage cover at reference sites, this may have contributed to observed differences particularly evident at Cheviot Beach (Fig. 6). Intertidal reefs with little to no canopy-forming algae may benefit from UAVs fitted with multispectral sensors to allow for greater spectral differentiation between similar morphologies.

Environmental drivers are known to underpin species distribution, particularly in the dynamic intertidal zone^42^. The stresses of low tide can cause desiccation and algal bleaching leading to competition for space along stratified shorelines^43, 44^. As intertidal assemblages change from low- to high-shore, variations in reef features, including elevation, may become important across a range of scales. Multi-scale geomorphic structure of the reef, captured by the UAV, allowed for testing of how these features influenced distribution of distinct algal and invertebrate assemblages. Combining fine-scale structural information with detailed assemblage data from on ground quadrat surveys revealed elevation and distance to seaward reef edge were the strongest physical drivers of observed species distributions. Elevation was the most important variable for algae and, a combination of elevation and distance to seaward reef edge was the most important for invertebrates. These results support previous studies revealing the importance of elevation for species of the intertidal environment^45, 46^. Low-shore intertidal reef has a longer inundation period reducing exposure to stressors such as desiccation and UV radiation. Understorey species further from the shoreline rely on canopy-forming species to ameliorate changeable environmental conditions^47, 48^. Previous studies have established the importance of intertidal canopy-forming macroalgae as autogenic ecosystem engineers facilitating a diverse intertidal biota^37, 39, 49, 50^. Canopy cover provides a bio-protective layer creating microhabitats that remain damper and cooler than exposed shores after the tides have receded^47, 51^ facilitating survival of obligate low-shore species in higher sections of the reef. The mitigating effects of the canopy cover demonstrates the relevance of UAV dominant algal classification across the reef, as these canopy-forming species are often correlated with biotic assemblages and can be used to assess ecosystem health^41, 50^. Two of the species in the turfing rhodophyte group, *C. implexa* and *C. officianalis*, were strongly associated with low elevation and seaward reef edge (Supplementary Fig. S3) which could explain the dependence on canopy-forming species to ameliorate the exposed conditions during low-tide, and thus the difference in the on-ground and UAV detection of these species due to canopy obstruction.

In this study, low cost UAV components were used that would be considered comparable to devices currently available to recreational drone users. This presents enormous opportunities to increase temporal data capture including seasonal trends through implementation of citizen science programs. Consumer multirotors are already capable of autonomous flight and are integrated seamlessly into photomosaicing software such as Pix4Dmapper (https://pix4d.com), with specialist applications allowing for cloud based data upload, processing and storage. Changes to UAV operation laws and certification requirements, in particular lightweight UAV platforms, around the world will provide new opportunities to utilise low-cost, remote sensing for monitoring programs. Combined with increased endurance through improvements in battery technology and miniaturisation of sensors, there is an opportunity for proliferation of UAV technology in natural resource management applications.

Intertidal reefs often exhibit a high degree of heterogeneity due to the dynamic environment, and it is important that the monitoring technique used can assess the varied habitat types across these reefs. The results of this study show that although UAVs may not be able to replace on-ground monitoring techniques on intertidal reefs, they provide a complementary data source giving a more comprehensive understanding of intertidal reef assemblages. UAV surveys could be undertaken more frequently to assess changes in the canopy-forming algal community as an indicator of reef health, with reductions in the number of quadrats required. Collecting data not only on the intertidal assemblages, but also on the fine-scale geomorphological features of the reef, such as topographical complexity of the reef at centimetre resolutions, will assist with future management strategies observing susceptibility of these habitats to pressures such as sea level rise.

## Electronic supplementary material

Supplementary Information

## References

[1] Costanza R (1997). The value of the world’s ecosystem services and natural capital. Nature.

[2] Dayton PK (1975). Experimental evaluation of ecological dominance in a rocky intertidal algal community. Ecological Monographs.

[3] Brown, H., Donnally, D., Woods, B. & Edmunds, M. Intertidal Reef Monitoring Program: Central Victoria Marine Protected Areas. *Parks Victoria Technical Series* (2014).

[4] Young M, Carr MH (2015). Application of species distribution models to explain and predict the distribution, abundance and assemblage structure of nearshore temperate reef fishes. Diversity and Distributions.

[5] Ierodiaconou D, Monk J, Rattray A, Laurenson L, Versace VL (2011). Comparison of automated classification techniques for predicting benthic biological communities using hydroacoustics and video observations. Continental Shelf Research.

[6] Zavalas R, Ierodiaconou D, Ryan D, Rattray A, Monk J (2014). Habitat classification of temperate marine macroalgal communities using bathymetric LiDAR. Remote Sens-Basel.

[7] Airoldi L (2003). Effects of patch shape in intertidal algal mosaics: roles of area, perimeter and distance from edge. Marine Biology.

[8] Underwood AJ (2000). Experimental ecology of rocky intertidal habitats: what are we learning?. J Exp Mar Biol Ecol.

[9] Anderson K, Gaston KJ (2013). Lightweight unmanned aerial vehicles will revolutionize spatial ecology. Frontiers in Ecology and the Environment.

[10] Feng Q, Liu J, Gong J (2015). UAV remote sensing for urban vegetation mapping using random forest and texture analysis. Remote Sens-Basel.

[11] Pereira, E. *et al*. Unmanned air vehicles for coastal and environmental research. *J Coastal Res*, 1557–1561 (2009).

[12] Allan BM, Ierodiaconou D, Nimmo DG, Herbert M, Ritchie EG (2015). Free as a drone: ecologists can add UAVs to their toolbox. Frontiers in Ecology and the Environment.

[13] Koh LP, Wich SA (2012). Dawn of drone ecology: low-cost autonomous aerial vehicles for conservation. Tropical Conservation Science.

[14] DeBusk, W. M. Unmanned aerial vehicle systems for disaster relief: tornado alley. AIAA Infotech@Aerospace 2010, *Infotech@Aerospace Conference*, doi:10.2514/6.2010-3506 (2010).

[15] Ierodiaconou, D., Schimel, A. C. G. & Kennedy, D. M. A new prespective of storm bite on sandy beaches using unmanned aerial vehicles. *Zeitschrift fur Geomorphologie Supplementary Issues*, doi:10.1127/zfg_suppl/2016/00247 (2016).

[16] Vega FA, Ramirez FC, Saiz MP, Rosua FO (2015). Multi-temporal imaging using an unmanned aerial vehicle for monitoring a sunflower crop. Biosyst Eng.

[17] Zahawi RA (2015). Using lightweight unmanned aerial vehicles to monitor tropical forest recovery. Biol Conserv.

[18] Quater, P. B., Grimaccia, F. & Masini, A. Airborne unmanned monitoring system for coastal erosion assessment. *Engineering Geology for Society and Territory*, Vol 4: *Marine and Coastal Processes*, 115–120, doi:10.1007/978-3-319-08660-6_22 (2014).

[19] Turner IL, Harley MD, Drummond CD (2016). UAVs for coastal surveying. Coastal Engineering.

[20] Casado MR, Gonzalez RB, Kriechbaumer T, Veal A (2015). Automated identification of river hydromorphological features using UAV high resolution aerial imagery. Sensors-Basel.

[21] Pérez-Ortiz M (2015). A semi-supervised system for weed mapping in sunflower crops using unmanned aerial vehicles and a crop row detection method. Appl Soft Comput.

[22] Chabot D, Bird DM (2015). Wildlife research and management methods in the 21st centuary: where do unmanned aircraft fit in?. Journal of Unmanned Vehicle Systems.

[23] Christiansen F, Dujon AM, Sprogis KR, Arnould JPY, Bejder L (2016). Noninvasive unmanned aerial vehicle provides estimates of the energetic cost of reproduction in humpback whales. Ecosphere.

[24] Hodgson, J. C., Baylis, S. M., Mott, R., Herrod, A. & Clarke, R. H. Precision wildlife monitoring using unmanned aerial vehicles. *Sci Rep-Uk* **6**, doi:10.1038/srep22574 (2016). 10.1038/srep22574 PMC4795075 26986721

[25] Goncalves JA, Henriques R (2015). UAV photogrammetry for topographic monitoring of coastal areas. Isprs J Photogramm.

[26] Guichard F, Bourget E, Agnard J-P (2000). High-resolution remote sensing of intertidal ecosystems: a low-cost technique to link scale-dependent patterns and processes. Limnology and Oceanography.

[27] Bryson, M., Johnson-Roberson, M., Murphy, R. J. & Bongiorno, D. Kite aerial photography for low-cost, ultra-high spatial resolution multi-spectral mapping of intertidal landscapes. *Plos One* **8**, doi:10.1371/journal.pone.0073550 (2013). 10.1371/journal.pone.0073550 PMC3777947 24069206

[28] Raoult, V. *et al*. GoPros as an underwater photogrammetry tool for citizen science. *Peerj* **4**, doi:10.7717/peerj.1960 (2016). 10.7717/peerj.1960 PMC4860335 27168973

[29] Longtin CM, Scrosati RA, Whalen GB, Garbary DJ (2009). Distribution of algal epiphytes across environmental gradients at different scales: intertidal elevation, host canopies, and host fronds. Journal of Phycology.

[30] Helmuth B, Mieszkowska N, Moore P, Hawkins SJ (2006). Living on the edge of two changing worlds: forecasting the responses of rocky intertidal ecosystems to climate change. Annual Review of Ecology, Evolution, and Systematics.

[31] R: A language for statistical computing (R Foundation for Statistical Computing, Vienna, Austria).

[32] Clarke KR (1993). Non-parametric multivariate analyses of changes in community structure. Aust J Ecol.

[33] Warwick RM, Clarke KR (1991). A comparison of some methods for analysing changes in benthic community structure. Journal of the Marine Biological Association of the United Kingdom.

[34] PERMANOVA+ for PRIMER: Guide to Software and Statistical Methods. PRIMER-E (Plymouth, UK, 2008).

[35] Kruskal JB (1964). Nonmetric multidimensional scaling: A numerical method. Psychometrika.

[36] Cortes R (2011). Effects of landscape metrics and land-use variables on macroinvertebrate communities and habitat characteristics. Limnetica.

[37] Lilley SA, Schiel DR, Underwood T (2006). Community effects following the deletion of a habitat-forming alga from rocky marine shores. Oecologia.

[38] Bertocci I (2010). Canopy-forming species mediate the effects of disturbance on macroalgal assemblages on Portuguese rocky shores. Marine Ecology Progress Series.

[39] Pocklington, J. B. *et al*. Disturbance alters ecosystem engineering by a canopy-forming alga. *Journal of the Marine Biological Association of the United Kingdom*, 1–12, doi:10.1017/S0025315416002009 (2017).

[40] Bellgrove A, Clayton MN, Quinn GP (2004). An integrated study of the temporal and spatial variation in the supply of propagules, recruitment and assemblages of intertidal macroalgae on a wave-exposed rocky coast, Victoria, Australia. J Exp Mar Biol Ecol.

[41] Irving AD, Connell SD (2006). Predicting understorey structure from the presence and composition of canopies: an assembly rule for marine algae. Oecologia.

[42] Scrosati RA, van Genne B, Heaven CS, Watt CA (2011). Species richness and diversity in different functional groups across environmental stress gradients: a model for marine rocky shores. Ecography.

[43] Thompson R, Crowe T, Hawkins S (2002). Rocky intertidal communities: past environmental changes, present status and predictions for the next 25 years. Environmental Conservation.

[44] Bellgrove A, McKenzie PF, McKenzie JL, Sfiligoj BJ (2010). Restoration of the habitat-forming fucoid alga *Hormosira banksii* at effluent-affected sites: competitive exclusion by coralline turfs. Marine Ecology Progress Series.

[45] Heaven CS, Scrosati RA (2008). Benthic community composition across gradients of intertidal elevation, wave exposure, and ice scour in Atlantic Canada. Marine Ecology Progress Series.

[46] Hollenbeck JP, Olsen MJ, Haig SM (2014). Using terrestrial laser scanning to support ecological research in the rocky intertidal zone. J Coast Conserv.

[47] Coombes MA, Naylor LA, Viles HA, Thompson RC (2013). Bioprotection and disturbance: seaweed, microclimatic stability and conditions for mechanical weathering in the intertidal zone. Geomorphology.

[48] Watt CA, Scrosati RA (2013). Bioengineer effects on understory species richness, diversity, and composition change along an environmental stress gradient: Experimental and mensurative evidence. Estuarine, Coastal and Shelf Science.

[49] Jenkins SR, Norton TA, Hawkins SJ (2004). Long term effects of Ascophyllum nodosum canopy removal on mid shore community structure. Journal of the Marine Biological Association of the United Kingdom.

[50] Schiel DR (2006). Rivets or bolts? When single species count in the function of temperate rocky reef communities. J Exp Mar Biol Ecol.

[51] Bellgrove A, McKenzie PF, Cameron H, Pocklington JB (2017). Restoring rocky intertidal communities: Lessons from a benthic macroalgal ecosystem engineer. Marine Pollution Bulletin.

