# Supplementary material for: Applications of unmanned aerial vehicles in intertidal reef monitoring
Source: Sci Rep. 2017 Aug 31;7:10259. doi: 10.1038/s41598-017-10818-9 (PMC5579233; doi:10.1038/s41598-017-10818-9)
Supplement: Supplementary file 1 — Supplementary Information [file 41598_2017_10818_MOESM1_ESM.pdf]

Supplementary Information

Applications of unmanned aerial vehicles in intertidal reef monitoring

Sarah L. Murfitt[1], Blake M. Allan[1,2], Alecia Bellgrove[1], Alex Rattray[1], Mary A. Young[1], *Daniel Ierodiaconou[1]

[1] Deakin University, School of Life and Environmental Sciences, Centre for Integrative Ecology, P.O. Box 423, Warrnambool, 3280, Victoria, Australia

[2]Victorian UAS Training, 57 Koroit-Woolsthrope Road, Koroit, 3282, Victoria, Australia

Corresponding author:

*Daniel Ierodiaconou iero@deakin.edu.au

Supplementary Tables and Figures

Supplementary Table S1

Table S1: Time, shown in minutes, to complete survey of all quadrats at sites. UAV flight: time taken to complete all flights required to capture entire survey area (active flight time at 10 m altitude); UAV virtual quadrats: time taken to extract quadrats from orthomosaic, import into Coral Point Count (v. 4.1), and complete 50 point count of algae; UAV total: UAV flight time and UAV virtual quadrats combined; On-ground quadrats: time taken to complete point count of algae and abundance counts of invertebrates (active count time and walking between quadrats at site). Shaded columns show total UAV and on-ground times used in statistical comparison.

	UAV flight	UAV virtual quadrats	UAV total	On-ground quadrats
Pickering Point	8	54	62	83
Shelly Beach	7	64	71	125
Point Lonsdale	36	45	81	150
Cheviot Beach	35	39	74	136
Ricketts Point	5	71	76	142
Halfmoon Bay	3	39	42	102
Mushroom Reef	12	37	49	129
West Flinders	4	50	54	143

Supplementary Table S2

Table S2: PERMANOVA of square-root transformed algal group percentage cover data observed by on-ground quadrats and UAV remote sensing across all eight sites. Bray-Curtis dissimilarity measure used. Statistically significant values marked with *

Source	df	MS	Pseudo-F	P(perm)
Region	3	85194	292.35	<0.001*
Park	1	29063	0.99	0.436
Method	1	6831.7	8.60	0.0102*
Region x Park	3	29452	101.07	<0.001*
Region x Method	3	794.44	2.73	0.005*
Park x Method	1	8041.6	55.49	<0.001*
Quadrat(Region x Park)	190	1604.9	5.51	<0.001*
Region x Park x Method	3	144.9	0.50	0.871
Residual	190	291.41		
Total	395			

Supplementary Figure S1

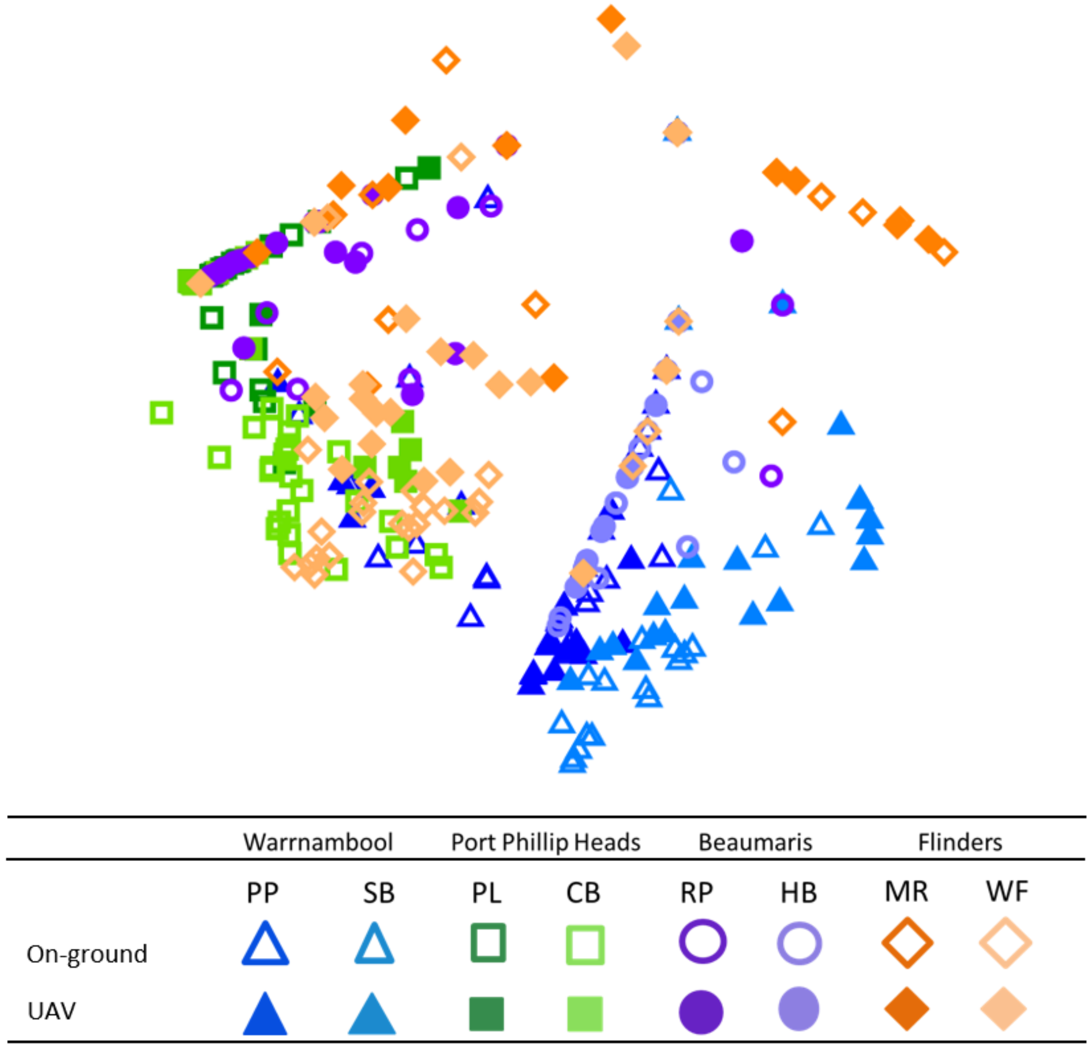

Figure S1: nMDS plot of percentage cover of four algal groups across Region (distinguished by symbols: triangles = Warrnambool, squares = Port Phillip Heads, circles = Beaumaris, diamonds = Flinders); Park (MPA = dark, reference = light colour); and Method (on-ground quadrat = open, UAV = closed). Sites: PP= Pickering Point, SB = Shelly Beach, PL = Point Lonsdale, CB = Cheviot Beach, RP = Ricketts Point, HB = Halfmoon Bay, MR = Mushroom Reef, WF = West Flinders. Data was square-root transformed. 2D stress 0.16.

Supplementary Figure S2

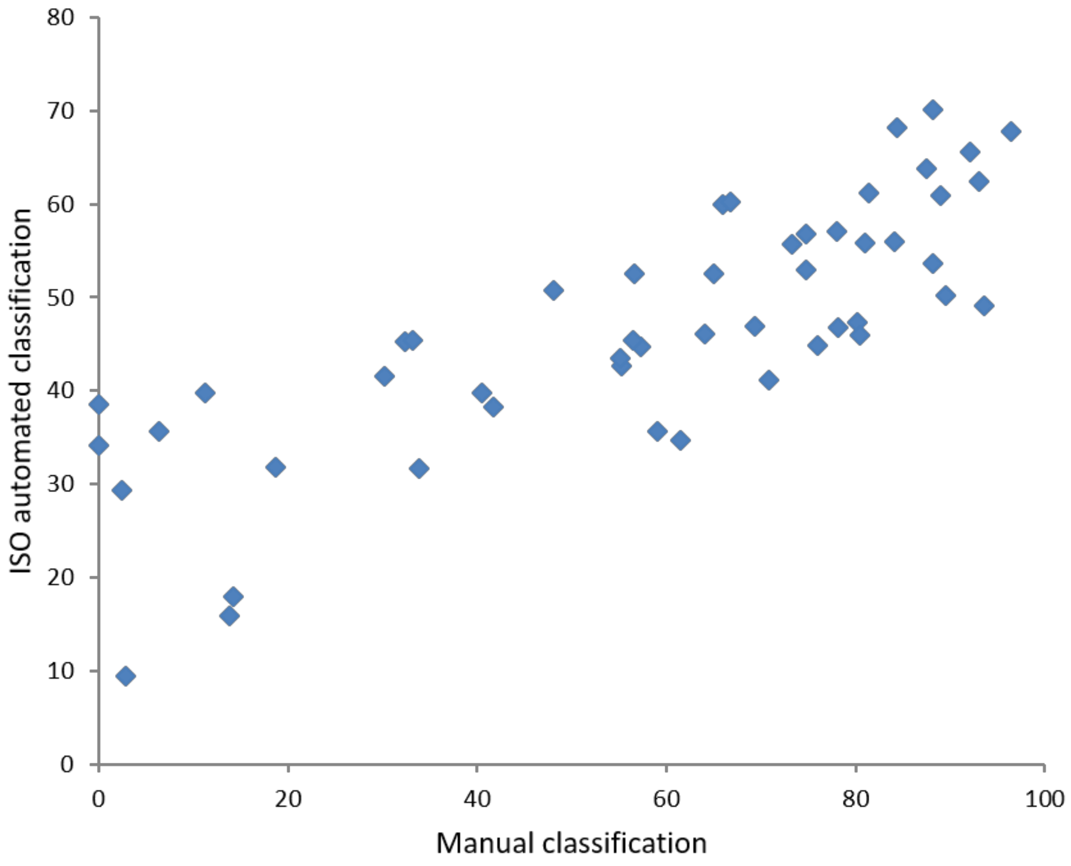

Figure S2: Scatterplot of manual and ISO automated estimates of percentage cover of *Hormosira banskii* in 50 quadrats at the Point Lonsdale site.

Supplementary Figure S3

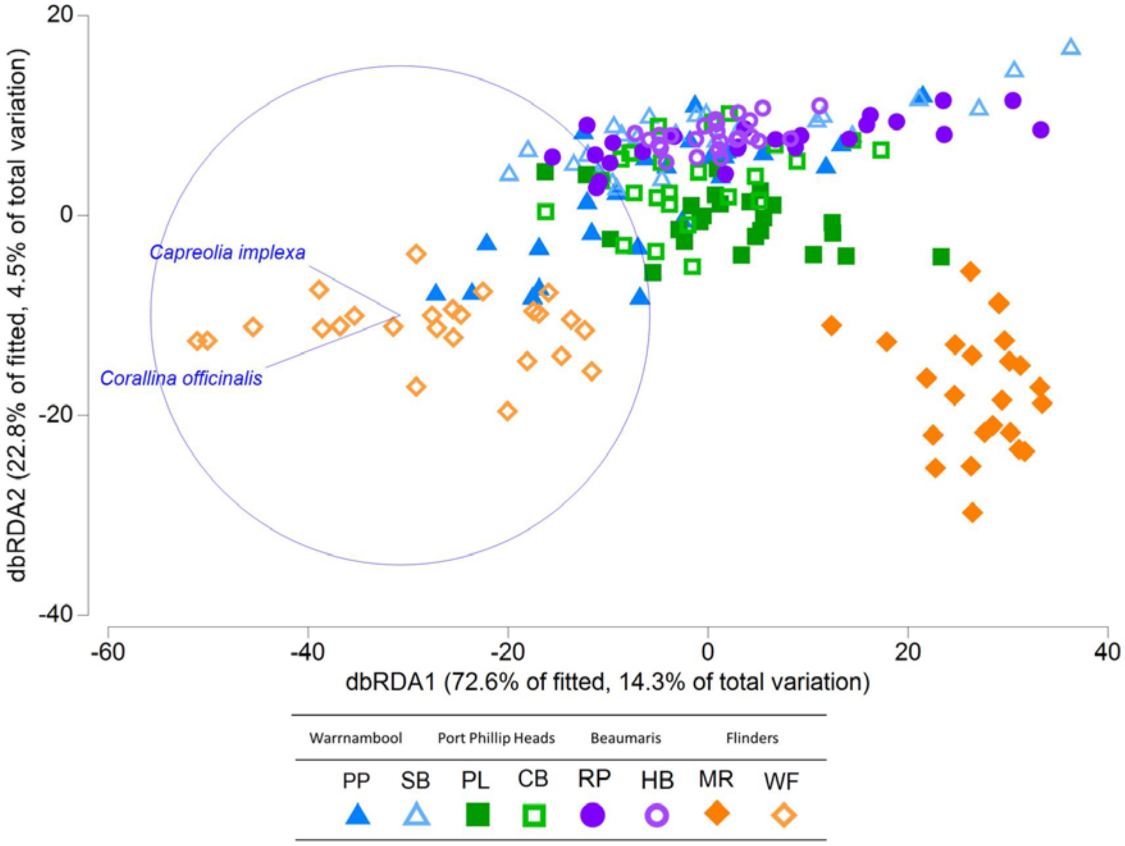

Figure S3: Distance-based redundancy analysis (dbRDA) ordination describing the relationship between derived environmental variables and algal percentage cover from on-ground quadrat DistLM classified by site. Vectors depict the effect of environmental variables influencing the presence of algal taxa with a Pearson's correlation of 0.4, with length of vector representing the strength of effect. Axes show percentage of variation for elevation (dbRDA1) and distance to seaward reef edge (dbRDA2). Sites: (PP= Pickering Point, SB = Shelly Beach, PL = Point Lonsdale, CB = Cheviot Beach, RP = Ricketts Point, HB = Halfmoon Bay, MR = Mushroom Reef, WF = West Flinders); Region distinguished by symbols: (triangles = Warrnambool, squares = Port Phillip Heads, circles = Beaumaris, diamonds = Flinders); Park (MPA = closed, reference = open).
